# Prevalence and Determinants of Computer Vision Syndrome Among Healthcare Providers in Al Baha, Saudi Arabia: A Cross-Sectional Study

**DOI:** 10.7759/cureus.85991

**Published:** 2025-06-14

**Authors:** Osama S AlGhamdi, Mohammed H Alshehri, Mahadi A Bashir

**Affiliations:** 1 Preventive Medicine Postgraduate Program, Department of Public Health Administration, Ministry of Health, Jeddah, SAU; 2 Department of Public Health, Ministry of Health, King Fahad General Hospital, Al Baha, SAU; 3 Department of Public Health Administration, Ministry of Health, Jeddah, SAU; 4 Department of General Surgery, Faculty of Medicine, Al Baha University, Al Baha, SAU

**Keywords:** asthenopia, computer vision syndrome (cvs), digital eye strain (des), dry eye disorder, practice of healthcare providers

## Abstract

Background

The widespread adoption of digital technology in healthcare settings has led to prolonged screen exposure among healthcare providers, increasing their risk of developing computer vision syndrome (CVS). CVS encompasses a range of ocular and extraocular symptoms that significantly impact well-being and productivity. This study aimed to investigate the prevalence and determinants of CVS among healthcare providers at King Fahad Hospital (KFH) in Al Baha, Saudi Arabia.

Methods

A hospital-based, cross-sectional study was conducted, involving 192 healthcare providers selected through stratified random sampling. After their consent, data were collected using a validated questionnaire assessing CVS symptoms, determinants, and preventive practices. Statistical analysis, including logistic regression, was performed to identify the factors associated with CVS.

Results

The prevalence of CVS was 59.4% (n=114), with pharmacists reporting the highest prevalence (n=16, 84.2%). Significant predictors of CVS included wearing eyeglasses (adjusted odds ratios (aOR)=3.76; 95% confidence interval (CI) 1.66-8.55; p=0.002), computer working time of three to six hours/day (aOR=3.40; 95% CI 1.20-9.67; p = 0.021), being a pharmacist (aOR=7.75; 95% CI 1.22-49.03; p=0.030), and being a nurse/midwife (aOR=3.22; 95% CI 1.01-10.24; p=0.047). Preventive practices, such as always taking regular breaks, were associated with reduced CVS prevalence (aOR=0.11; 95% CI 0.02-0.80; p=0.029). Headache, eye burning, and dryness were the most commonly reported symptoms, while ergonomic practices were inconsistently implemented.

Conclusion

CVS is a prevalent occupational health issue among healthcare providers at KFH, driven by demographic and occupational factors. Effective preventive measures are essential to mitigate the impact of CVS.

## Introduction

The use of digital technology has become an essential part of people's daily lives; it has solved many problems and brought a great advantage (e.g., time-saving nature) to many disciplines, especially in medical practice. Nevertheless, some disadvantages have also resulted from the heavy adoption of such technologies and significant dependence on them [1]. For instance, in medical education, this adoption of digital technology has increased screen time for medical students due to their greater need to fulfill educational requirements in terms of studying, research, and clinical practice [2]. This demand has continued during career paths, potentially increasing their risk of developing negative outcomes.

Computer vision syndrome (CVS), also known as digital eye strain or visual fatigue, is one of the disadvantages of digital technology overuse and a negative outcome. It is defined by the American Optometric Association as "a group of ocular and visual symptoms that results from prolonged usage of computers, tablets, e-readers, and cell phones which causes increased stress to near vision in particular" [3]. CVS includes a set of symptoms ranging from ocular symptoms such as eyestrain, blurred vision, and dry eyes and extraocular symptoms like headaches, neck pain, or even shoulder pain, altogether affecting normal well-being and productivity [4,5].

On occupational sittings, CVS is considered an ergonomics-based related healthcare issue [6]. It affects workers' quality of life and, hence, their performance and productivity. For instance, in 2007, a study was conducted among 665 computer users and concluded that eye symptoms were weakly but significantly linked to lower overall quality of life [7].

A literature review related to CVS identified two recent systematic reviews reaching broadly similar conclusions (published in 2023 and 2024, respectively) [8,9]. These comprehensive reviews captured the most relevant studies, minimizing the need for further extensive literature searches related to the research question.

The first review (conducted in 2022) incorporated 45 studies and undertook a meta-analysis to estimate the pooled prevalence of CVS. Their analysis yielded a prevalence of 66% (95% CI: 59-74) [8]. Office workers (e.g., government employees, bank workers) and students were predominantly the subjects of most included studies. Only two studies (out of 45 included studies) were among healthcare providers. The first study, conducted in Saudi Arabia in 2021, was the first to investigate CVS among radiologists there and concluded that CVS is prevalent among radiologists, with 50.5% of the participants reporting having at least one symptom [10]. The second study, conducted in Spain, adopted a broader approach by encompassing various healthcare specialties. It held a similar groundbreaking status, being the first to explore CVS among healthcare providers globally. Similarly, they concluded that CVS is prevalent among healthcare providers (56.75%) [11]. In addition to these two studies, another study was included in this review, which examined CVS among staff members of the medical college at Taibah University in Saudi Arabia [12]. Despite a primary focus on education and knowledge dissemination within the healthcare landscape, they remain designated as healthcare providers. In this study, they concluded a higher prevalence of CVS (81.3%) [12]. 

Conversely, the latter review (published in 2024) was more robust and comprehensive. It included 103 studies with a higher estimated pooled prevalence of 69% (95% CI: 62.3-75.3) [9]. All previously mentioned studies in the first systematic review were also included in this review. In addition to them, we also have included a few other studies conducted in healthcare settings. For instance, another study examined the prevalence of CVS among radiologists in Saudi Arabia [13]. Contrary to the previous study (which was conducted among radiologists who only reside in Eastern Saudi Arabia) [10], researchers conducted a nationwide survey that included all radiologists in Saudi Arabia. Despite this, they concluded a similar but slightly higher prevalence of 65.4% (95% CI: 60.8 to 70) [13]. One study explored the prevalence and associated risks of CVS in a tertiary care hospital in Central India. Even though they had expanded their aim into assessing knowledge of CVS through the pre-test/post-test methodology, they only included office workers in their study, hindering its representativeness. Nevertheless, their analysis yielded an estimated prevalence of 78% [14]. In addition to the two systematic reviews, a recently published study from Saudi Arabia examined CVS among healthcare providers working in primary healthcare facilities [15]. Although the study did not report an overall prevalence of CVS, it documented a varied prevalence of eye symptoms, ranging from 3.0% to 73.3% [15].

Regarding the determinants of CVS, the literature review identified a third systematic review (which was carried out by the same investigating team in the first review) [16]. In this review, the main focus was on exploring factors associated with CVS. Several significant associations were identified. For instance, female gender, improper posture during electronic device usage, non-work-related screen time, the absence of regular break periods, prolonged visual display terminal exposure, close viewing distance, and deficiencies in ergonomic practices were all found to be positively correlated with an increased likelihood of developing CVS [16]. In addition, the previously mentioned second review demonstrated a sex disparity in CVS prevalence, with females exhibiting a higher proportion compared to males (71.4% and 61.8%. respectively) [9]. Moreover, a geographical variation is observed, with seemingly elevated prevalence in Africa and Asia compared to Latin America and Europe. In addition, they reported an association between contact lens usage and an increased prevalence of CVS [9]. In general, similar conclusions were reached by earlier studies that reviewed CVS and supported such findings [17,18].

In exploring preventive measures, the literature findings can be summarized into two main aspects. The first aspect is practicing better work ergonomics and modifying the work environment. For instance, environment modification can be achieved by controlling environmental factors, such as maintaining balanced humidity, reducing air pollutants, and ensuring proper ambient lighting conditions with reduced glare (e.g., using screens and blinds over light sources and windows) [19,20]. Regarding work ergonomics, recommendations favor adjusting the screen location to be below eye level and setting the screen brightness at low and proper levels using screen guards and blue filters [21,22]. Additionally, maintaining a proper seating position and body posture is recommended [5]. The second aspect focuses on controlling screen time and implementing protective ocular practices, such as frequent blinking and taking regular breaks (e.g., following the 20-20-20 rule). Correcting refractive errors and using anti-glare eyewear are also recommended [23].

In summary, the scarcity of research on CVS among healthcare providers is evident. Therefore, the aim of the study is to investigate the prevalence and determinants of CVS among healthcare providers in King Fahad Hospital (KFH) at Al Baha, Saudi Arabia, and to assess the extent to which preventive measures, such as seating position, viewing distance, daily exposure time, breaks, and frequent blinking, are being implemented by them, enhancing scientific knowledge and guiding the development of interventions to improve their visual health and overall well-being.

## Materials and methods

Study design and population

This is a hospital-based, cross-sectional study. The target population for this study was healthcare providers working in KFH in Al Baha province, Saudi Arabia. Al Baha province is located in the southwestern region of Saudi Arabia, with a population of 339,174 (as of 2022) [24]. Al Baha city serves as the capital of the province. KFH stands as the largest healthcare facility in the province, with a bed capacity of 404 beds and offering a wide range of medical services [25].

Sampling and sample size

The sampling technique employed in this study involved obtaining a comprehensive list of all healthcare providers, including their emails, contact numbers, and occupations, from KFH's human resources department (HR). This list served as the sampling frame for participant selection. To ensure a representative sample, a stratified random sampling technique was adopted. Population strata were defined based on the latest statistical yearbook published by the Ministry of Health in Saudi Arabia and are as follows: Physicians/Dentists, Nurses/Midwives, Pharmacists, and Allied Health Personnel [26].

The proportion of each stratum within the target population was determined based on up-to-date HR records. After that, a simple random selection of participants within each stratum was conducted using a computer-based random number generator [27]. This approach provided each employee with an equal chance of being selected for participation in the study.

Selected participants were contacted via email as the primary mode of communication. They received an email that included detailed information about the study and a link to the questionnaire. A follow-up reminder was sent within the sampling timeframe to mitigate the non-response rate.

This study included all healthcare providers working in KFH. It only excluded those who have active or chronic ocular disease (e.g., conjunctivitis or glaucoma), any specific eyelid disorders (e.g., blepharitis), or uncorrected refractive errors (e.g., uncorrected myopia). The questionnaire design ensured these criteria were met.

Calculating the sample size was based on a prevalence estimate of 81.3% from a recent local study [12], as it represents the closest geographical and population context to our target population. Using a total population of 968 (based on HR records), an error margin of 5%, and a confidence level of 95%, the following Cochran's formula [28] was used:

$n= \tfrac{N\cdot z^2\cdot \hat{p}(1-\hat{p})}{\varepsilon^2 (N-1)+z^2\cdot\hat{p}(1-\hat{p})}$

The calculated sample size is 188.35, rounded up to 189. A 10% increase was added to the initial calculation to account for anticipated non-response rates, resulting in a final sample size of 208.

Data sources and collection

A structured, web-based, self-administered questionnaire was developed following a comprehensive literature review and expert consultation. The questionnaire consists of three sections as follows.

Demographic and Occupational Data Section

This section was dedicated to collecting relevant demographic data such as age, gender, nationality, and marital status. It also includes data regarding occupational characteristics such as specialty, working institution, and years of experience. 

Computer Vision Syndrome Questionnaire (CVS-Q)

A questionnaire developed and validated by Seguí et al. (2015) [29], consisting of 16 items that measure CVS symptoms in terms of frequency, intensity, and ultimately diagnosis of CVS. The frequency and intensity were graded on a scale from 0 to 2 (respectively, 0=never, 1=occasionally, 2=often or always, and 1=moderate, 2=intense), and then the frequency and intensity were multiplied, and CVS was diagnosed in participants scoring 6 or higher (scale 0-32).

Determinants and Preventive Measures and Practice Section

A pretested structured questionnaire was used to assess determinants and preventive measures practice related to CVS, such as seating position, viewing distance, daily exposure time, breaks, and frequent blinking. This questionnaire was adapted from a previous study by Assefa et al. (2017) [30].

Statistical analysis

Data were analyzed using IBM SPSS for Statistics, version 27 (IBM Corp, Armonk, NY). All required assumptions were checked, and normality tests were performed. Descriptive statistics were used to summarize the data by estimating mean (±standard deviation) for continuous variables and frequency (and percentage) for categorical variables. The significance of associations was determined using the chi-square test or Fisher’s exact test when assumptions were not met. Binary logistic regression models were employed to calculate crude odds ratios (cOR) of possible predictors. A multiple logistic regression model was employed to adjust for confounding predictors and derive final estimations and adjusted odds ratios (aOR). The p-value of significance was set at (<0.05) and a 95% confidence interval (CI).

Ethics approval and consent to participate

Ethical approval was obtained from the KFH Institutional Review Board (IRB) at the Ministry of Health, Al Baha, Saudi Arabia (IRB number: KFH/IRB04072024/3). Informed consent was obtained from participants, providing them with comprehensive information about the study and their rights to withdraw at any time without any obligations.

## Results

Of the 208 healthcare providers invited to participate, 204 responses were received (initial response rate 98.1%). After excluding 12 responses due to reported eye conditions (one acute eye disease and 11 chronic eye diseases), 192 responses were included in the final analysis (92.3% completion rate).

Demographic and occupational distributions

The participants' ages ranged from 23 to 54 years, with a mean age of 32.1 years (SD=6.8). Among the 192 participants, 60 (31.3%) were in the 26-30 years age group, representing the majority, followed by 52 (27.1%) in the 31-35 years age group. Gender distribution was nearly even, with 100 (52.1%) of them being women. Regarding marital status, 99 (51.6%) were single. In terms of nationality, 141 (73.4%) were Saudi. Non-smokers comprised the majority, as 151 (78.6%) fell into this category. Eyeglasses were worn by 80 (41.7%), with the highest usage among physicians, as 32 (56.1%) of them reported wearing eyeglasses (Table 1).

**Table 1:** Demographic characteristics across occupational categories of studied participants.

Variables	Physician, n (%)	Dentist, n (%)	Pharmacist, n (%)	Nurse/Midwife, n (%)	Allied Health Personnel, n (%)	Total, N (%)
Frequencies	57 (29.7%)	8 (4.2%)	19 (9.9%)	60 (31.3%)	48 (25.0%)	192 (100%)
Age						
Mean (SD)	33.75 (7.61)	28.00 (1.00)	24.00 (0.89)	34.43 (6.81)	32.86 (9.16)	32.11 (6.80)
Median (range)	31 (26-49)	28 (27-29)	28 (27-29)	33.50 (24-54)	30 (23-52)	31 (23-54)
20-25	1 (1.8%)	0 (0.0%)	16 (84.2%)	3 (5.0%)	11 (22.9%)	31 (16.1%)
26-30	23 (40.4%)	3 (37.5%)	3 (15.8%)	10 (16.7%)	21 (43.8%)	60 (31.3%)
31-35	15 (26.3%)	2 (25.0%)	0 (0.0%)	30 (50.0%)	5 (10.4%)	52 (27.1%)
36-40	8 (14.0%)	3 (37.5%)	0 (0.0%)	13 (21.7%)	6 (12.5%)	30 (15.6%)
>40	10 (17.5%)	0 (0.0%)	0 (0.0%)	4 (6.7%)	5 (10.4%)	19 (9.9%)
Gender						
Male	37 (64.9%)	7 (87.5%)	6 (31.6%)	19 (31.7%)	23 (47.9%)	92 (47.9%)
Female	20 (35.1%)	1 (12.5%)	13 (68.4%)	41 (68.3%)	25 (52.1%)	100 (52.1%)
Nationality						
Saudi	41 (71.9%)	6 (75.0%)	19 (100.0%)	29 (48.3%)	46 (95.8%)	141 (73.4%)
Non-Saudi	16 (28.1%)	2 (25.0%)	0 (0.0%)	31 (51.7%)	2 (4.2%)	51 (26.6%)
Marital status						
Married	32 (56.1%)	3 (37.5%)	3 (15.8%)	32 (53.3%)	23 (47.9%)	93 (48.4%)
Single	25 (43.9%)	5 (62.5%)	16 (84.2%)	28 (46.7%)	25 (52.1%)	99 (51.6%)
Smoking status						
Non-Smoker	41 (71.9%)	8 (100.0%)	17 (89.5%)	52 (86.7%)	33 (68.8%)	151 (78.6%)
Smoker	16 (28.1%)	0 (0.0%)	2 (10.5%)	8 (13.3%)	15 (31.3%)	41 (21.4%)
Wear eyeglasses						
No	25 (43.9%)	8 (100.0%)	9 (47.4%)	41 (68.3%)	29 (60.4%)	112 (58.3%)
Yes	32 (56.1%)	0 (0.0%)	10 (52.6%)	19 (31.7%)	19 (39.6%)	80 (41.7%)
Years of service						
<5	32 (56.1%)	3 (37.5%)	17 (89.5%)	13 (21.7%)	26 (54.2%)	91 (47.4%)
5-10	12 (21.1%)	0 (0.0%)	2 (10.5%)	24 (40.0%)	12 (25.0%)	50 (26.0%)
>10	13 (22.8%)	5 (62.5%)	0 (0.0%)	23 (38.3%)	10 (20.8%)	51 (26.6%)
Working time on Computer (hours/day)						
<3	9 (15.8%)	4 (50.0%)	2 (10.5%)	10 (16.7%)	15 (31.3%)	40 (20.8%)
3-6	29 (50.9%)	3 (37.5%)	8 (42.1%)	21 (35.0%)	16 (33.3%)	77 (40.1%)
>6	19 (33.3%)	1 (12.5%)	9 (47.4%)	29 (48.3%)	17 (35.4%)	75 (39.1%)

The sample primarily included 60 (31.3%) nurses/midwives, mostly women, 41 (68.3%), followed by 57 (29.7%) physicians, where men dominated, 34 (64.9%). Allied health personnel were 45 (25%), while pharmacists were 19 (9.9%), and dentists were only eight (4.2%). In terms of experience, 91 (47.4%) had less than five years of experience, with the rest split equally between the remaining categories, as 50 (26%) indicated five to 10 years of experience and 51 (26.6%) reported more than 10 years of experience. Regarding computer use, the most common usage time was three to six hours reported by 77 (40.1%) participants, followed by extended use (>six hours), as noted by 75 (39.1%) participants. Using computers for less than three hours daily was reported by 40 (20.8%) participants (Table 1).

Prevalence of CVS

CVS was present among 114 (59.4%) participants, with significant differences across occupations (p=0.012). Pharmacists had the highest prevalence, as 16 (84.2%) had CVS, and dentists had the lowest, as only three (37.5%) had CVS. Age differences were only significant among individuals aged 20-25 (p=0.037), with pharmacists showing the highest rate (87.5%). Women had a significantly higher prevalence (n=70, 70.0%) than men (n=44, 47.8%; p=0.002). Among physicians, those who were single were more affected (n=17, 68.0%) than married individuals (n=13, 40.6%; p=0.040), with similar variations across occupational groups (p=0.035). While Saudi nationals had a higher overall CVS prevalence (n=82, 72.7%), differences between Saudi and non-Saudi participants were not statistically significant, though borderline variation existed across occupations (p=0.049) (Table 2).

**Table 2:** Prevalence of CVS among participants based on demographics and occupational groups. Note: p-values were calculated using Chi-square test. Fisher’s exact test used when expected counts were less than 5 in more than 20% of cells. Abbreviations: CVS: Computer Vision Syndrome, p: p-value ^a^Differences between variables. ^b^Differences between occupational groups. ^c^Chi-square test. ^d^Fisher’s exact test. ^e^No statistics computed as variable is a constant.

Variables	Physician, n (%)	p^a^	Dentist, n (%)	p^a^	Pharmacist, n (%)	p^a^	Nurse/Midwife, n (%)	p^a^	Allied Health, n (%)	p^a^	Total, N (%)	p^a^	p^b^
Frequencies	30 (52.6%)		3 (37.5%)		16 (84.2%)		38 (70.0%)		23 (47.9%)		114 (59.4%)		0.012^c^
Age		0.642^d^		0.143^d^		0.422^d^		0.341^d^		0.774^d^		0.109^c^	
20-25	0 (0.0%)		0 (0.0%)		14 (87.5%)		2 (45.5%)		5 (45.5%)		21 (67.7%)		0.037^d^
26-30	14 (60.9%)		0 (0.0%)		2 (66.7%)		8 (52.4%)		11 (52.4%)		35 (58.3%)		0.165^d^
31-35	7 (46.7%)		2 (100.0%)		0 (0.0%)		21 (60.0%)		3 (60.0%)		33 (63.5%)		0.396^d^
36-40	5 (62.5%)		1 (33.3%)		0 (0.0%)		10 (50.0%)		3 (50.0%)		19 (63.3%)		0.489^d^
>40	4 (40.0%)		0 (0.0%)		0 (0.0%)		1 (20.0%)		1 (20.0%)		6 (31.6%)		0.823^d^
Gender		0.792^c^		1.000^d^		0.222^d^		0.046^c^		0.081^c^		0.002^c^	
Male	19 (51.4%)		3 (42.9%)		4 (66.7%)		10 (34.8%)		8 (34.8%)		44 (47.8%)		0.598^d^
Female	11 (55.0%)		0 (0.0%)		12 (92.3%)		32 (60.0%)		15 (60.0%)		70 (70.0%)		0.032^d^
Nationality		0.402^c^		1.000^d^		null^e^		0.464^c^		1.000^d^		0.567^c^	
Saudi	23 (56.1%)		2 (33.3%)		16 (84.2%)		19 (47.8%)		22 (47.8%)		82 (58.2%)		0.049^c^
Non-Saudi	7 (43.8%)		1 (50.0%)		0 (0.0%)		23 (50.0%)		1 (50.0%)		32 (62.7%)		0.108^d^
Marital status		0.040^c^		1.000^d^		0.422^d^		0.821^c^		0.571^c^		0.125^c^	
Married	13 (40.6%)		1 (33.3%)		2 (66.7%)		22 (52.2%)		12 (52.2%)		50 (53.8%)		0.181^d^
Single	17 (68.0%)		2 (40.0%)		14 (87.5%)		20 (44.0%)		11 (44.0%)		64 (64.6%)		0.035^c^
Smoking status		0.804^c^		null^e^		1.000^d^		0.247^c^		0.401^c^		0.596^c^	
Non-Smoker	22 (53.7%)		3 (37.5%)		14 (82.4%)		35 (54.5%)		18 (54.5%)		92 (60.9%)		0.112^c^
Smoker	8 (50.0%)		0 (0.0%)		2 (100.0%)		7 (33.3%)		5 (33.3%)		22 (53.7%)		0.041^d^
Wear eyeglasses		0.026^c^		null^e^		0.087^d^		0.303^c^		0.263^c^		0.005^c^	
No	9 (36.0%)		3 (37.5%)		6 (66.7%)		27 (41.4%)		12 (41.4%)		57 (50.9%)		0.075^d^
Yes	21 (65.6%)		0 (0.0%)		10 (100.0%)		15 (57.9%)		11 (57.9%)		57 (71.3%)		0.081^c^
Years of service		0.435^c^		0.196^d^		1.000^d^		0.355^c^		0.100^c^		0.185^c^	
<5	19 (59.4%)		0 (0.0%)		14 (82.4%)		7 (42.3%)		11 (42.3%)		51 (56.0%)		0.029^c^
5-10	6 (50.0%)		0 (0.0%)		2 (100.0%)		18 (83.3%)		10 (83.3%)		36 (72.0%)		0.279^d^
>10	5 (38.5%)		3 (60.0%)		0 (0.0%)		17 (20.0%)		2 (20.0%)		27 (52.9%)		0.017^d^
Working time on computer (hours/day)		0.107^d^		0.679^d^		1.000^d^		0.007^c^		0.347^d^		<.001^c^	
<3	2 (22.2%)		1 (25.0%)		2 (100.0%)		3 (33.3%)		5 (33.3%)		13 (32.5%)		0.415^d^
3-6	18 (62.1%)		2 (66.7%)		7 (87.5%)		15 (50.0%)		8 (50.0%)		50 (64.9%)		0.434^d^
>6	10 (52.6%)		0 (0.0%)		7 (77.8%)		24 (58.8%)		10 (58.8%)		51 (68.0%)		0.070^d^

Smoking showed significant differences across occupational groups (p=0.041), but not between smokers and non-smokers overall. Eyeglass use was strongly linked to CVS (p=0.005), with wearers having a higher prevalence (71.3%) than non-wearers (50.9%). This trend was significant among physicians (p=0.026). Years of service did not influence CVS prevalence overall, but variations were significant in those with less than five years (p=0.029) and >10 years of service (p=0.017). Computer usage had the strongest association with CVS (p<0.001); prevalence was highest (n=51, 68.0%) among those using computers for over six hours daily and lowest (n=13, 32.5%) in those using them for less than three hours. Nurses/midwives also showed significant differences (p=0.007) (Table 2).

Symptoms of CVS

The most frequently reported symptoms among participants were headache (n=128, 66.7%), eye burning (n=124, 64.6%), and dryness (n=117, 60.9%), while the least common were colored halos around objects (n=30, 15.6%), double vision (n=30, 15.6%), and heavy eyelids (n=47, 24.5%). Among those diagnosed with CVS, eye burning (n=99, 86.8%), headache (n=98, 86.0%), and dryness (n=97, 85.1%) were most prevalent, whereas for those without CVS, headache (n=30, 38.5%) was the most common, followed by eye burning (n=25, 32.1%) and dryness (n=20, 25.6%). The least reported symptoms among CVS cases were double vision (n=27, 23.7%), colored halos (n=28, 24.6%), and heavy eyelids (n=43, 37.7%), while in non-CVS participants, excessive blinking (n=2, 2.6%), colored halos (n=2, 2.6%), and double vision (n=3, 3.8%) were the least noted. All CVS-Q symptoms were significantly different across groups (p<0.001) as CVS diagnosis was based on total CVS-Q scores (Table 3).

**Table 3:** Distribution of CVS symptoms among participants based on CVS-Q. ^a^Chi-square test. ^b^Fisher’s exact test. Abbreviations: CVS: computer vision syndrome, CVS-Q: Computer Vision Syndrome Questionnaire.

Symptoms (Intensity)	Have CVS (%), n=78	No CVS (%), n=114	Total (%), N=192	Test value	p-value
1. Eye burning				68.838^a^	<.001^a^
Never	15 (13.2%)	53 (67.9%)	68 (35.4%)		
Occasionally (Moderate)	59 (51.8%)	24 (30.8%)	83 (43.2%)		
Occasionally (Intense)	22 (19.3%)	1 (1.3%)	23 (12.0%)		
Often or always (Moderate)	13 (11.4%)	0 (0.0%)	13 (6.8%)		
Often or always (Intense)	5 (4.4%)	0 (0.0%)	5 (2.6%)		
2. Eye itching				65.766^b^	<.001^b^
Never	23 (20.2%)	60 (76.9%)	83 (43.2%)		
Occasionally (Moderate)	55 (48.2%)	16 (20.5%)	71 (37.0%)		
Occasionally (Intense)	19 (16.7%)	1 (1.3%)	20 (10.4%)		
Often or always (Moderate)	10 (8.8%)	1 (1.3%)	11 (5.7%)		
Often or always (Intense)	7 (6.1%)	0 (0.0%)	7 (3.6%)		
3. Feeling of a foreign body				80.439^b^	<.001^b^
Never	34 (29.8%)	72 (92.3%)	106 (55.2%)		
Occasionally (Moderate)	45 (39.5%)	6 (7.7%)	51 (26.6%)		
Occasionally (Intense)	19 (16.7%)	0 (0.0%)	19 (9.9%)		
Often or always (Moderate)	12 (10.5%)	0 (0.0%)	12 (6.3%)		
Often or always (Intense)	4 (3.5%)	0 (0.0%)	4 (2.1%)		
4. Tearing				55.668^a^	<.001^a^
Never	31 (27.2%)	63 (80.8%)	94 (49.0%)		
Occasionally (Moderate)	46 (40.4%)	12 (15.4%)	58 (30.2%)		
Occasionally (Intense)	19 (16.7%)	3 (3.8%)	22 (11.5%)		
Often or always (Moderate)	18 (15.8%)	0 (0.0%)	18 (9.4%)		
Often or always (Intense)	0 (0.0%)	0 (0.0%)	0 (0.0%)		
5. Excessive blinking				73.334^b^	<.001^b^
Never	46 (40.4%)	76 (97.4%)	122 (63.5%)		
Occasionally (Moderate)	35 (30.7%)	2 (2.6%)	37 (19.3%)		
Occasionally (Intense)	24 (21.1%)	0 (0.0%)	24 (12.5%)		
Often or always (Moderate)	6 (5.3%)	0 (0.0%)	6 (3.1%)		
Often or always (Intense)	3 (2.6%)	0 (0.0%)	3 (1.6%)		
6. Eye redness				42.211^a^	<.001^a^
Never	35 (30.7%)	59 (75.6%)	94 (49.0%)		
Occasionally (Moderate)	41 (36.0%)	16 (20.5%)	57 (29.7%)		
Occasionally (Intense)	23 (20.2%)	3 (3.8%)	26 (13.5%)		
Often or always (Moderate)	14 (12.3%)	0 (0.0%)	14 (7.3%)		
Often or always (Intense)	1 (0.9%)	0 (0.0%)	1 (0.5%)		
7. Eye pain				43.452^b^	<.001^b^
Never	57 (50.0%)	73 (93.6%)	130 (67.7%)		
Occasionally (Moderate)	36 (31.6%)	5 (6.4%)	41 (21.4%)		
Occasionally (Intense)	13 (11.4%)	0 (0.0%)	13 (6.8%)		
Often or always (Moderate)	5 (4.4%)	0 (0.0%)	5 (2.6%)		
Often or always (Intense)	3 (2.6%)	0 (0.0%)	3 (1.6%)		
8. Heavy eyelids				30.939^b^	<.001^b^
Never	71 (62.3%)	74 (94.9%)	145 (75.5%)		
Occasionally (Moderate)	26 (22.8%)	3 (3.8%)	29 (15.1%)		
Occasionally (Intense)	11 (9.6%)	0 (0.0%)	11 (5.7%)		
Often or always (Moderate)	1 (0.9%)	1 (1.3%)	2 (1.0%)		
Often or always (Intense)	5 (4.4%)	0 (0.0%)	5 (2.6%)		
9. Dryness				72.943^a^	<.001^a^
Never	17 (14.9%)	58 (74.4%)	75 (39.1%)		
Occasionally (Moderate)	42 (36.8%)	14 (17.9%)	56 (29.2%)		
Occasionally (Intense)	24 (21.1%)	5 (6.4%)	29 (15.1%)		
Often or always (Moderate)	14 (12.3%)	1 (1.3%)	15 (7.8%)		
Often or always (Intense)	17 (14.9%)	0 (0.0%)	17 (8.9%)		
10. Blurred vision				31.654^b^	<.001^b^
Never	53 (46.5%)	67 (85.9%)	120 (62.5%)		
Occasionally (Moderate)	42 (36.8%)	9 (11.5%)	51 (26.6%)		
Occasionally (Intense)	12 (10.5%)	1 (1.3%)	13 (6.8%)		
Often or always (Moderate)	5 (4.4%)	1 (1.3%)	6 (3.1%)		
Often or always (Intense)	2 (1.8%)	0 (0.0%)	2 (1.0%)		
11. Double vision				13.893^b^	<.001^b^
Never	87 (76.3%)	75 (96.2%)	162 (84.4%)		
Occasionally (Moderate)	20 (17.5%)	3 (3.8%)	23 (12.0%)		
Occasionally (Intense)	3 (2.6%)	0 (0.0%)	3 (1.6%)		
Often or always (Moderate)	4 (3.5%)	0 (0.0%)	4 (2.1%)		
Often or always (Intense)	0 (0.0%)	0 (0.0%)	0 (0.0%)		
12. Difficulty focusing for near vision				25.251^b^	<.001^b^
Never	69 (60.5%)	72 (92.3%)	141 (73.4%)		
Occasionally (Moderate)	29 (25.4%)	6 (7.7%)	35 (18.2%)		
Occasionally (Intense)	7 (6.1%)	0 (0.0%)	7 (3.6%)		
Often or always (Moderate)	5 (4.4%)	0 (0.0%)	5 (2.6%)		
Often or always (Intense)	4 (3.5%)	0 (0.0%)	4 (2.1%)		
13. Increased sensitivity to light				36.806^b^	<.001^b^
Never	53 (46.5%)	69 (88.5%)	122 (63.5%)		
Occasionally (Moderate)	33 (28.9%)	7 (9.0%)	40 (20.8%)		
Occasionally (Intense)	19 (16.7%)	2 (2.6%)	21 (10.9%)		
Often or always (Moderate)	4 (3.5%)	0 (0.0%)	4 (2.1%)		
Often or always (Intense)	5 (4.4%)	0 (0.0%)	5 (2.6%)		
14. Colored halos around objects				17.974^b^	<.001^b^
Never	86 (75.4%)	76 (97.4%)	162 (84.4%)		
Occasionally (Moderate)	21 (18.4%)	2 (2.6%)	23 (12.0%)		
Occasionally (Intense)	5 (4.4%)	0 (0.0%)	5 (2.6%)		
Often or always (Moderate)	1 (0.9%)	0 (0.0%)	1 (0.5%)		
Often or always (Intense)	1 (0.9%)	0 (0.0%)	1 (0.5%)		
15. Feeling that sight is worsening				40.373^b^	<.001^b^
Never	53 (46.5%)	69 (88.5%)	122 (63.5%)		
Occasionally (Moderate)	33 (28.9%)	8 (10.3%)	41 (21.4%)		
Occasionally (Intense)	18 (15.8%)	0 (0.0%)	18 (9.4%)		
Often or always (Moderate)	5 (4.4%)	1 (1.3%)	6 (3.1%)		
Often or always (Intense)	5 (4.4%)	0 (0.0%)	5 (2.6%)		
16. Headache				54.832^a^	<.001^a^
Never	16 (14.0%)	48 (61.5%)	64 (33.3%)		
Occasionally (Moderate)	47 (41.2%)	22 (28.2%)	69 (35.9%)		
Occasionally (Intense)	32 (28.1%)	4 (5.1%)	36 (18.8%)		
Often or always (Moderate)	7 (6.1%)	4 (5.1%)	11 (5.7%)		
Often or always (Intense)	12 (10.5%)	0 (0.0%)	12 (6.3%)		

Occupationally, pharmacists reported dryness most frequently (n=18, 94.7%), followed by headache (n=14,73.7%). Headache was the most common symptom among physicians (n=37, 64.9%) and nurses/midwives (n=47, 78.3%), with eye burning as the second most reported (n=33, 57.9% and n=46, 76.7%, respectively). In allied health personnel, eye burning (n=27, 56.3%) was the dominant symptom, followed by headache (n=25, 52.1%). Dentists primarily reported eye redness (n=6, 75.0%) and eye burning (n=5, 62.5%) (Table 4).

**Table 4:** CVS symptoms prevalence by occupation Note: counts are of participants who reported having the symptom, disregarding the intensity and frequency. Abbreviations: CVS: Computer vision syndrome.

Symptom	Physicians, n (%)	Dentists, n (%)	Pharmacists, n (%)	Nurses/Midwives, n (%)	Allied Health Personnel, n (%)	Total, n (%)
1. Eye burning	33 (57.9%)	5 (62.5%)	13 (68.4%)	46 (76.7%)	27 (56.3%)	124 (64.6%)
2. Eye itching	27 (47.4%)	5 (62.5%)	13 (68.4%)	40 (66.7%)	24 (50.0%)	109 (56.8%)
3. Feeling of a foreign body	25 (43.9%)	2 (25.0%)	13 (68.4%)	26 (43.3%)	20 (41.7%)	86 (44.8%)
4. Tearing	22 (38.6%)	5 (62.5%)	11 (57.9%)	38 (63.3%)	22 (45.8%)	98 (51.0%)
5. Excessive blinking	19 (33.3%)	3 (37.5%)	7 (36.8%)	24 (40.0%)	17 (35.4%)	70 (36.5%)
6. Eye redness	23 (40.4%)	6 (75.0%)	11 (57.9%)	39 (65.0%)	19 (39.6%)	98 (51.0%)
7. Eye pain	16 (28.1%)	1 (12.5%)	8 (42.1%)	22 (36.7%)	15 (31.3%)	62 (32.3%)
8. Heavy eyelids	9 (15.8%)	0 (0.0%)	2 (10.5%)	24 (40.0%)	12 (25.0%)	47 (24.5%)
9. Dryness	33 (57.9%)	2 (25.0%)	18 (94.7%)	40 (66.7%)	24 (50.0%)	117 (60.9%)
10. Blurred vision	21 (36.8%)	1 (12.5%)	10 (52.6%)	18 (30.0%)	22 (45.8%)	72 (37.5%)
11. Double vision	6 (10.5%)	0 (0.0%)	7 (36.8%)	6 (10.0%)	11 (22.9%)	30 (15.6%)
12. Difficulty focusing for near vision	18 (31.6%)	0 (0.0%)	6 (31.6%)	13 (21.7%)	14 (29.2%)	51 (26.6%)
13. Increased sensitivity to light	20 (35.1%)	0 (0.0%)	7 (36.8%)	26 (43.3%)	17 (35.4%)	70 (36.5%)
14. Colored halos around objects	14 (24.6%)	0 (0.0%)	5 (26.3%)	3 (5.0%)	8 (16.7%)	30 (15.6%)
15. Feeling that sight is worsening	22 (38.6%)	2 (25.0%)	11 (57.9%)	17 (28.3%)	18 (37.5%)	70 (36.5%)
16. Headache	37 (64.9%)	5 (62.5%)	14 (73.7%)	47 (78.3%)	25 (52.1%)	128 (66.7%)

Preventive measures and ergonomic practices

**Figure 1:**
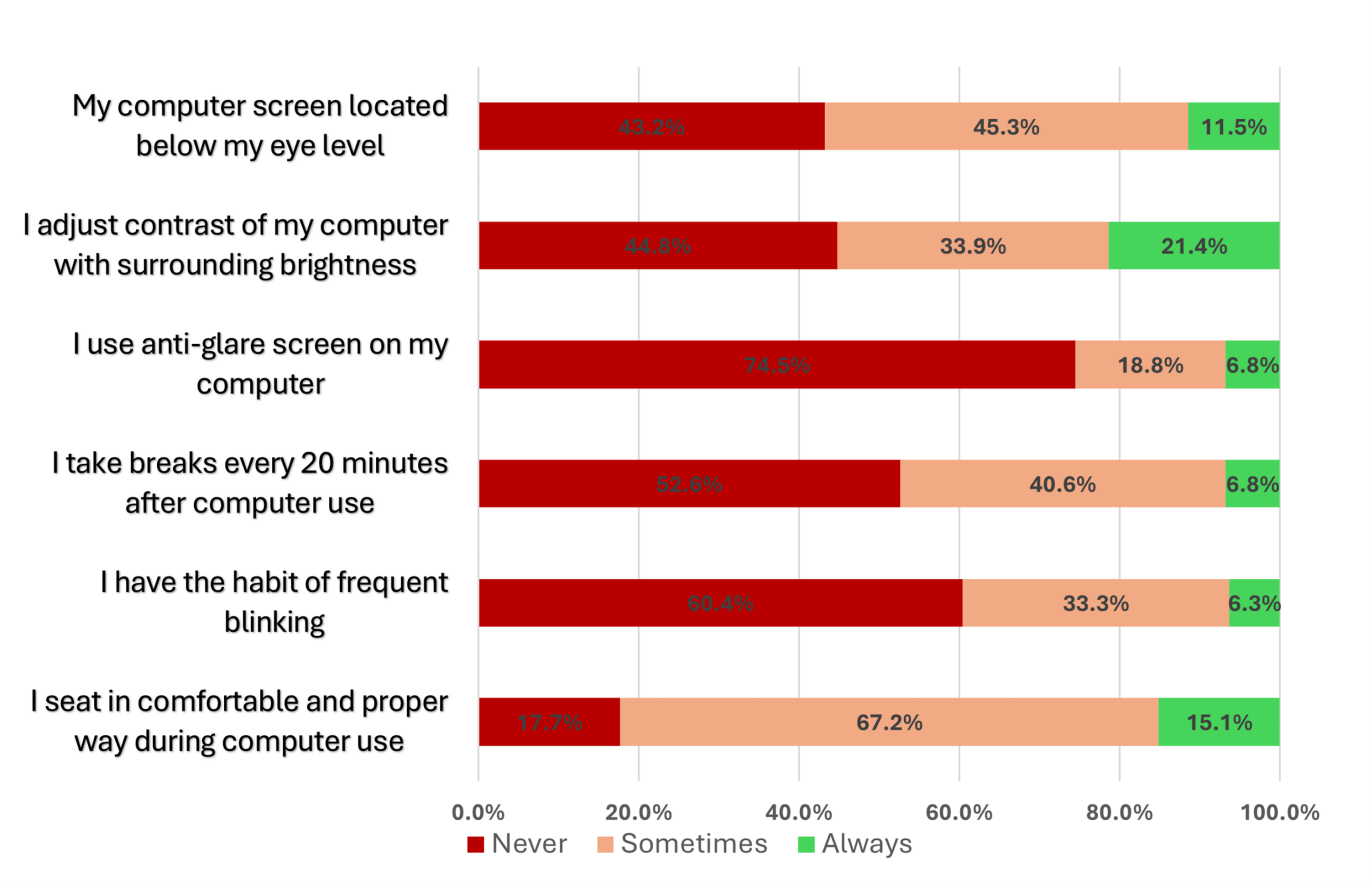
Implementation of ergonomics and preventive measures. *Implementation Gap*=difference between “Always” and target 100%.

Among participants, 41 (21.4%) reported “always” adjusting computer brightness to match their surroundings, making it the most common ergonomic practice. This was followed by maintaining a comfortable seating position (n=29, 15.1%) and positioning the screen below eye level (n=22, 11.5%). These practices varied by CVS status. For instance, those affected by CVS were slightly more likely to locate the computer screen below eye level (n=14, 12.3%) compared to those without CVS (n=8, 10.3%). However, overall ergonomic practices were more frequently reported by non-CVS participants, though most differences were not statistically significant. Two exceptions were noted: proper seating posture, which was significantly more common among non-CVS participants (n=18, 23.1%) than those with CVS (n=11, 9.6%; p=0.033), and taking breaks every 20 minutes, reported significantly more often by non-CVS participants (n=10, 12.8%) compared to those with CVS (n=3, 2.6%; p=0.022) (Table 5, Figure 1).

**Table 5:** Preventive measures and ergonomics practices among participants based on their CVS condition. ^a^Chi-square test. *Significant p-value < 0.05. Abbreviations: CVS: computer vision syndrome.

Practice		Frequency	Have CVS (%), n=78	No CVS (%), n=114	Total (%), N=192	Test value^a^	p-value^a^
I sit in a comfortable and proper way during computer use						6.851	0.033*
		Never	20 (17.5%)	14 (17.9%)	34 (17.7%)		
		Sometimes	83 (72.8%)	46 (59.0%)	129 (67.2%)		
		Always	11 (9.6%)	18 (23.1%)	29 (15.1%)		
I have the habit of frequently blinking						0.390	0.823
		Never	67 (58.8%)	49 (62.8%)	116 (60.4%)		
		Sometimes	40 (35.1%)	24 (30.8%)	64 (33.3%)		
		Always	7 (6.1%)	5 (6.4%)	12 (6.3%)		
I take breaks every 20 minutes after computer use						7.629	0.022*
		Never	63 (55.3%)	38 (48.7%)	101 (52.6%)		
		Sometimes	48 (42.1%)	30 (38.5%)	78 (40.6%)		
		Always	3 (2.6%)	10 (12.8%)	13 (6.8%)		
I use an anti-glare screen on my computer						3.061	0.216
		Never	89 (78.1%)	54 (69.2%)	143 (74.5%)		
		Sometimes	20 (17.5%)	16 (20.5%)	36 (18.8%)		
		Always	5 (4.4%)	8 (10.3%)	13 (6.8%)		
I adjust the contrast of my computer with the surrounding brightness						0.386	0.824
		Never	53 (46.5%)	33 (42.3%)	86 (44.8%)		
		Sometimes	38 (33.3%)	27 (34.6%)	65 (33.9%)		
		Always	23 (20.2%)	18 (23.1%)	41 (21.4%)		
My computer screen is located below my eye level						1.172	0.557
		Never	52 (45.6%)	31 (39.7%)	83 (43.2%)		
		Sometimes	48 (42.1%)	39 (50.0%)	87 (45.3%)		
		Always	14 (12.3%)	8 (10.3%)	22 (11.5%)		

Predictors of CVS and associated factors

Bivariate analysis identified several factors significantly associated with a higher probability of CVS, including being a woman (cOR=2.55; p=0.002), wearing eyeglasses (cOR=2.39; p=0.005), working on a computer for three to six hours/day (cOR=3.85; p=0.001) or more than six hours/day (cOR=4.41; p<0.001), and being a pharmacist (cOR=4.80; p=0.022). Conversely, participants aged 41 years or older (cOR=0.22; p=0.015) and those who “always” took breaks every 20 minutes (cOR=0.18; p=0.013) had significantly lower CVS probability (Table 6).

**Table 6:** Bivariate analysis assessing the association between CVS and demographics, occupational and ergonomic factors. Note: p-values were calculated using binary logistic regression model for each variable. *Significant p-value < 0.05. Abbreviations: CVS: computer vision syndrome, cOR: crude odds ratio, CI: confidence interval.

Variables	cOR	95% CI	p-value
Age (years)			
20-25	1.00		
26-30	0.67	0.27-1.66	0.383
31-35	0.83	0.32-2.12	0.693
36-40	0.82	0.29-2.37	0.717
>40	0.22	0.06-0.75	0.015*
Gender			
Male	1.00		
Female	2.55	1.41-4.60	0.002*
Nationality			
Saudi	1.00		
Non-Saudi	1.21	0.63-2.34	0.568
Marital status			
Married	1.00		
Single	1.57	0.88-2.80	0.126
Smoking status			
Non-Smoker	1.00		
Smoker	0.74	0.37-1.49	0.402
Wear eyeglasses			
No	1.00		
Yes	2.39	1.30-4.40	0.005*
Occupational Factors:			
Years of service (years)			
<5	1.00		
5-10	2.02	0.96-4.24	0.064
>10	0.88	0.44-1.76	0.722
Working time on Computer (hours/day)			
<3	1.00		
3-6	3.85	1.71-8.65	0.001*
>6	4.41	1.94-10.03	<.001>*
Occupation			
Physicians	1.00		
Dentists	0.54	0.12-2.48	0.428
Pharmacists	4.80	1.26-18.30	0.022*
Nurses/Midwives	2.10	0.98-4.48	0.055
Allied Health Personnel	0.82	0.38-1.77	0.630
Behavioral factors			
I sit in a comfortable and proper way during computer use			
Never	1.00		
Sometimes	1.26	0.58-2.73	0.533
Always	0.43	0.16-1.18	0.101
I have the habit of frequently blinking			
Never	1.00		
Sometimes	1.22	0.65-2.28	0.535
Always	1.02	0.31-3.42	0.969
I take breaks every 20 minutes after computer use			
Never	1.00		
Sometimes	0.97	0.53-1.77	0.909
Always	0.18	0.05-0.70	0.013*
I use an anti-glare screen on my computer			
Never	1.00		
Sometimes	0.76	0.36-1.59	0.463
Always	0.38	0.12-1.22	0.104
I adjust the contrast of my computer with the surrounding brightness			
Never	1.00		
Sometimes	0.88	0.45-1.69	0.694
Always	0.80	0.37-1.69	0.553
My computer is screen located below my eye level			
Never	1.00		
Sometimes	0.73	0.40-1.36	0.323
Always	1.04	0.39-2.77	0.932

A multiple logistic regression model adjusting for confounding variables confirmed that wearing eyeglasses (aOR=3.76; p=0.002), working on a computer for three to six hours/day (aOR=3.40; p=0.021), being a pharmacist (aOR=7.75; p=0.030), and being a nurse/midwife (aOR=3.22; p=0.047) remained significant risk factors. However, “always” taking breaks every 20 minutes (aOR=0.11; p=0.029) was the only significant protective factor. The model was statistically significant (𝑥²=61.8; p<0.001), explained 37.2% of CVS variation (Nagelkerke R²), fit the data well (Hosmer and Lemeshow test, p=0.392), and correctly classified 75% of participants (Table 7).

**Table 7:** Multiple logistic regression model of CVS predictors (demographics, occupational and ergonomic factors). *Significant p-value < 0.05. Abbreviations: CVS: computer vision syndrome, aOR: adjusted odds ratio, CI: confidence interval, 𝑥^2^: chi-square. *Model summary*: Hosmer and Lemeshow test, p=0.392; Omnibus test:  𝑥^2^=61.8, p=<.001; accuracy=75%, Nagelkerke R^2^: 0.372

Variables	aOR	95% CI	p-value
Age (years)			
20-25	1.00		
26-30	1.34	0.34-5.36	0.677
31-35	1.60	0.31-8.32	0.578
36-40	2.64	0.29-23.85	0.386
>40	1.07	0.09-12.17	0.957
Gender			
Male	1.00		
Female	2.14	0.92-4.95	0.076
Nationality			
Saudi	1.00		
Non-Saudi	1.65	0.57-4.79	0.359
Marital status			
Married	1.00		
Single	1.51	0.57-4.00	0.407
Smoking status			
Non-Smoker	1.00		
Smoker	1.27	0.47-3.44	0.631
Wear eyeglasses			
No	1.00		
Yes	3.76	1.66-8.55	0.002*
Occupational Factors:			
Years of service (years)			
<5	1.00		
5-10	1.45	0.51-4.18	0.487
>10	0.68	0.14-3.35	0.639
Working time on Computer (hours/day)			
<3	1.00		
3-6	3.40	1.20-9.67	0.021*
>6	2.28	1.94-10.03	0.125
Occupation			
Physicians	1.00		
Dentists	1.74	0.21-14.25	0.605
Pharmacists	7.75	1.22-49.03	0.030*
Nurses/Midwives	3.22	1.01-10.24	0.047*
Allied Health Personnel	1.60	0.52-4.88	0.411
Behavioural Factors:			
I seat in comfortable and proper way during computer use			
Never	1.00		
Sometimes	1.35	0.50-3.65	0.559
Always	0.56	0.15-2.11	0.389
I have the habit of frequent blinking			
Never	1.00		
Sometimes	1.83	0.76-4.41	0.179
Always	3.40	0.63-18.21	0.153
I take breaks every 20 minutes after computer use			
Never	1.00		
Sometimes	1.24	0.54-2.85	0.610
Always	0.11	0.02-0.80	0.029*
I use anti-glare screen on my computer			
Never	1.00		
Sometimes	0.35	0.12-1.02	0.055
Always	0.69	0.10-4.96	0.715
I adjust contrast of my computer with surrounding brightness			
Never	1.00		
Sometimes	1.09	0.41-2.92	0.857
Always	1.22	0.39-3.87	0.731
My computer screen located below my eye level			
Never	1.00		
Sometimes	1.08	0.42-2.75	0.880
Always	2.19	0.51-9.33	0.290

## Discussion

This study is not the first to assess the prevalence of CVS, the practice of ergonomics, and preventive measures among healthcare providers in Saudi Arabia, as a recently published study addressed these topics [15]. Despite reporting a wide range of prevalence (3.0%-73.3%) for various eye symptoms, that study did not determine the overall prevalence of CVS [15]. Therefore, this study is the first to establish the overall prevalence of CVS among healthcare providers in Saudi Arabia and to quantify its relationship with demographic, occupational, and behavioral factors.

The overall prevalence of CVS in the current study is 59.4%, which aligns with the findings of a Spanish study conducted in similar settings (56.75%) [11]. This result is also consistent with the overall trends observed in two recent systematic reviews conducted in 2023 and 2024, which reported prevalences of 66.0% and 69.0%, respectively [8,9]. Similar findings were reported in studies conducted among students in China and Peru, with prevalence rates of 57.0% and 58.3%, respectively [31,32]. This concordance could be attributed to similarities in research methodologies, equal gender distributions, and the predominance of younger participants in the studied populations.

In contrast, a study conducted among university medical staff in Saudi Arabia reported a higher prevalence (81.3%) despite using the same measurement tool (CVS-Q) [12]. This discrepancy may be explained by the older age of participants in that study (mean age: 47.08±8.06 years) and the academic nature of their profession, which involves extensive computer use [12]. Similarly, higher prevalence rates were reported in other professions, such as information technology (IT) professionals and bankers, with rates of 82.4% and 84.8%, respectively [33,34].

Regarding CVS symptoms, headache, eye burning, and eye dryness were the most commonly reported symptoms, consistent with many studies conducted in similar settings [10,13]. Conversely, double vision was among the least reported symptoms, in agreement with findings from previous studies [10,35]. The alignment in symptom distribution across studies conducted in Saudi Arabia may be influenced by the arid desert environment, as suggested by Zalat et al [12].

When examining factors associated with CVS, age was not significantly associated with CVS in the current study. However, older participants aged 35-40 years had more than double the likelihood of having CVS compared to those aged 20-25 years (aOR=2.64; 95% CI 0.29-23.85; p=0.386). This non-significant finding is supported by a few previous studies [36,37]. For instance, Alhassan et al. examined CVS among radiologists and reported no significant difference in CVS median scores between age groups younger than 35 years (median=7) and older than 35 years (median=8; p=0.787) [13]. In contrast, Artime Ríos et al., in their novel study using artificial intelligence techniques, have identified age as a factor related to CVS and reported that the average age of those with CVS (45.92±10.06 years) was lower than the average age of those without CVS (47.12±11.31 years) [38].

The discrepancy in findings may be attributed to the differing effects of age-related factors. Younger individuals often experience higher CVS prevalence due to increased exposure to digital devices, whereas older individuals may develop CVS as a result of age-related visual changes and reduced adaptability to prolonged screen use. However, it is unclear whether asthenopia during computer use is associated with age as stated by Rosenfield [18]. In addition, different methodologies in measurement and statistical adjustments could contribute to such conflicting findings.

Regarding gender, women were more than twice as likely to have CVS than men in the current study (aOR=2.14; 95% CI 0.92-4.95; p=0.076), although this association was not statistically significant. This could be attributed to the multivariate adjustment in the model, which included occupations as a factor. This adjustment likely diminished the significance of gender by attributing it to occupations where women constituted the majority of the workforce, such as nurses and pharmacists, as will be discussed later on.

A statistically significant association was observed between wearing eyeglasses and an increased likelihood of developing CVS (aOR=3.76; 95% IC 1.66-8.55; p=0.002). This finding aligns with prior research demonstrating a significant relationship between eyeglass use and CVS symptoms among radiologists in Saudi Arabia, which attributed this to prolonged digital device use and optical adjustments for screen clarity [10]. However, Kamal and Abd El-Mageed found no significant association between eyeglass use and CVS prevalence among bank employees, suggesting that occupational and environmental factors might mediate this relationship [34].

Working for three to six hours per day on a computer was associated with an increased probability of CVS (aOR=3.40; p=0.021). This finding is consistent with studies by Tesfaye et al., which demonstrated that longer durations of computer use significantly elevated CVS prevalence among academic staff in Ethiopia [35], and Alenazi et al. who reported similar associations among healthcare professionals in Saudi Arabia [15]. However, Zalat et al. argued that while longer screen time was associated with CVS, the relationship could be moderated by individual ergonomic practices and the quality of workstation setups, which were not fully explored in the current study [12].

The occupational role was another critical determinant of CVS in the current study, with pharmacists (aOR=7.75; 95% CI 1.22-49.03; p=0.030) and nurses/midwives (aOR = 3.22; 95% CI 1.01-10.24; p=0.047) being particularly affected. Similar findings were reported by Artime-Ríos et al., who highlighted that nurses experienced the highest CVS prevalence among healthcare providers due to prolonged exposure to video display terminals and task-related stress [11,38]. However, disagreement arises from Kamal et al., who found that office-based roles in banking environments, despite involving extensive screen use, exhibited lower CVS prevalence compared to healthcare settings, possibly due to better ergonomic interventions in office setups [34]. The current study finding could be attributed to the fact that women were representing the majority of participants in these occupations as supported by Artime Ríos et al. [38].

Encouragingly, the current study highlighted that always taking breaks every 20 minutes significantly reduced the likelihood of CVS (aOR=0.11; p=0.029). This finding reinforces recommendations from Zalat et al., who emphasized that frequent breaks are critical in mitigating CVS symptoms among university staff [12]. However, Alhasan and Aalam reported that while taking breaks helped alleviate symptoms, its effectiveness was inconsistent among radiologists, likely due to the intensity and continuity of diagnostic tasks [13].

Despite the robust design and insightful results, the current study's limitations should be acknowledged. The cross-sectional nature of the study precludes establishing causal relationships, and self-reported data may introduce recall bias. Moreover, a small sample size and concise location may hinder the generalizability of findings and limit further statistical adjustments. In addition, several predictors such as computer use outside work, other visual display terminal use, and quality of workstation setups were not included for feasibility and logistics reasons. Therefore, future studies should consider longitudinal designs to explore temporal relationships between CVS and its risk factors.

## Conclusions

This study examines CVS prevalence of 59.4% (n=114) among Saudi healthcare providers and its links to demographic, occupational, and behavioral factors. These findings align with global trends, highlighting extended screen time, job roles, and eyewear as key contributors, while frequent breaks help prevent CVS. Despite limitations, the study emphasizes the need for ergonomic interventions. Further research is required to confirm these findings across different populations and work environments.
